# Progressive left cervical mass in an infant diagnosed as neuroglial heterotopia: A case report

**DOI:** 10.4102/sajr.v30i1.3542

**Published:** 2026-07-31

**Authors:** Alaipreet Kaur, Sameer Vyas, Atul Jain, Hemlata Jangir

**Affiliations:** 1 Department of Radiodiagnosis, Division of Neuroimaging and Interventional Neuroradiology, Post Graduate Institute of Medical Education and Research, Chandigarh; 2 Department of Histopathology, Post Graduate Institute of Medical Education and Research, Chandigarh

**Keywords:** pediatrics, neuroglial, heterotopia, parapharyngeal region, cystic hygroma, cystic neck mass

## Abstract

Neuroglial heterotopia is a congenital developmental anomaly characterised by mature glial tissue located outside the cranial cavity or spinal canal, without intracranial communication. A 12-month-old girl presented with an enlarging left cervical mass and worsening respiratory symptoms. MRI demonstrated a trans-spatial, multiseptated cystic lesion in the left parotid and submandibular spaces, extending medially into the parapharyngeal space with upper airway compression. No intracranial communication was identified. The postoperative specimen revealed mature glial tissue interspersed within fibroconnective stroma, confirming neuroglial heterotopia.

**Contribution:**

This case highlights the importance of considering neuroglial heterotopia in the differential diagnosis for congenital or progressive cystic neck masses in infants. Although MRI plays a central role in defining the lesion extent, a definitive diagnosis requires histopathological confirmation.

## Introduction

Neuroglial heterotopia is an uncommon developmental anomaly characterised by mature glial tissue located outside the cranial cavity or spinal canal. Unlike encephalocoele, it lacks communication with the subarachnoid space or intracranial contents. Historically, these lesions were described as ‘nasal gliomas’ when occurring in the nasal region; however, they are better regarded as heterotopic congenital rests of neuroectodermal tissue rather than true neoplasms.^1,2^

Several embryologic mechanisms have been proposed, including sequestration of herniated brain tissue after closure of a skull defect, displacement of neuroectodermal cells during embryogenesis, and aberrant migration of olfactory bulb-derived glial tissue.^3^ Most lesions arise in the nasal cavity, while extra-nasal locations such as the scalp, orbit, palate, tongue, middle ear and pharynx are rare.^4,5,6,7^ Parapharyngeal neuroglial heterotopia is particularly unusual and often poses a diagnostic challenge because its clinical and radiologic features overlap with those of lymphatic malformation, teratoma and other congenital neck masses.

## Ethical considerations

This case report describes standard clinical management and does not involve experimental procedures or interventions. This article followed all ethical standards for research, and informed consent was obtained from the parents for data and publication.

## Case presentation

A 12-month-old girl with an antenatally diagnosed left-sided cervical mass, presented with progressive enlargement and swelling of the mass, and worsening respiratory symptoms. She had been delivered at another hospital and had previously undergone tracheostomy elsewhere because of airway compromise.

**FIGURE 1:**
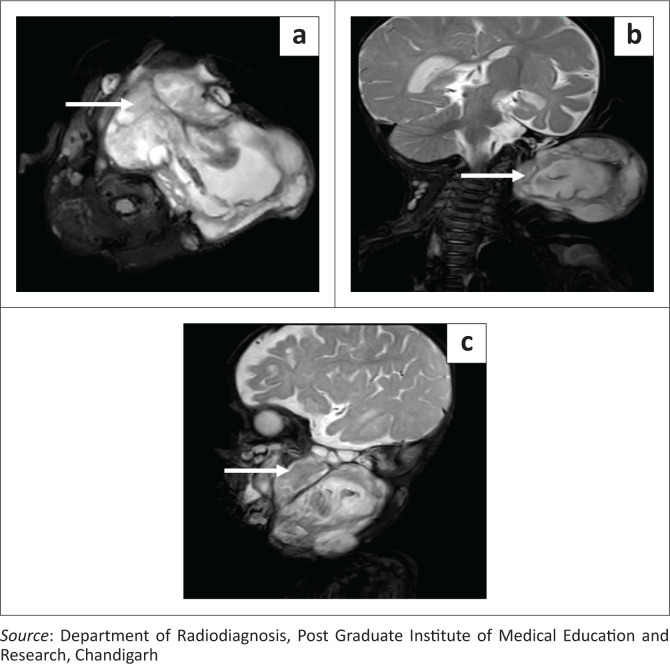
Axial (a), coronal (b), and sagittal (c) T2-weighted MR images demonstrate a large, trans-spatial, solid-cystic lesion involving the left cervical region. The cystic components are T2 hyperintense, while the eccentric solid component (arrows) demonstrates T2 iso to hypointense signal similar to grey matter. The lesion extends medially into the parapharyngeal space and laterally into the parotid and submandibular spaces, resulting in focal outward bulging of the overlying skin contour.

**FIGURE 2:**
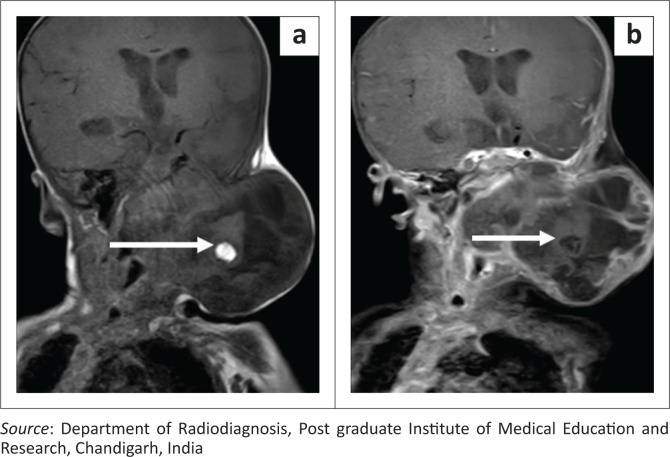
Coronal T1-weighted pre-contrast image (a) demonstrates a predominantly T1-hypointense cystic component, with the solid component iso to hypointense in signal to brain grey matter. A small central focus of intrinsic T1 hyperintensity (arrow) is noted within the lesion. On the coronal post-contrast fat-suppressed T1-weighted image (b), the solid component and septa reveal enhancement, while the central T1-hyperintense focus (arrow) demonstrates signal suppression, likely representing fat.

On examination, a soft, non-tender mass was palpable on the left side of the neck. MRI revealed a large, lobulated, trans-spatial, solid and cystic lesion involving the left side of the neck. Medially, the lesion extended into the parapharyngeal space, producing significant compression and rightward displacement of the nasopharynx, oropharynx and larynx, with marked luminal narrowing of the airway. Laterally, it involved the parotid and submandibular spaces, and there was a focal outward contour bulge of the overlying skin. The left submandibular gland was compressed and displaced inferomedially. Superiorly, the lesion reached the skull base, but no definite intracranial extension was identified. The cystic component of the lesion was T2 hyperintense, and the eccentric solid component was iso to hypointense in signal to brain parenchyma grey matter on T2 (Figure 1). It was T1 hypointense (Figure 2) with the solid component showing a T1 iso to hypointense signal to brain parenchyma. A small focus of T1 hyperintensity was seen within this lesion, which was hypointense on the T1 post-contrast fat-suppressed sequence, likely representing a fatty component. Post-contrast enhancement was seen within the solid component of the lesion. Based on the imaging appearance, cystic hygroma or lymphatic malformation were considered the leading radiologic differential diagnoses.

**FIGURE 3:**
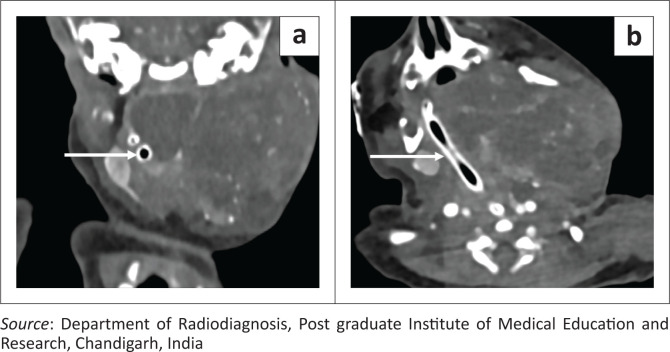
Coronal (a) and axial contrast-enhanced CT images (b) demonstrate a hypodense cystic lesion in the left neck, extending medially into the nasopharynx and causing marked airway compromise. Laterally, the lesion produces focal contour bulging of the overlying skin and involves the parotid and submandibular spaces. The tracheostomy tube is compressed and displaced by the mass (*arrows*).

**FIGURE 4:**
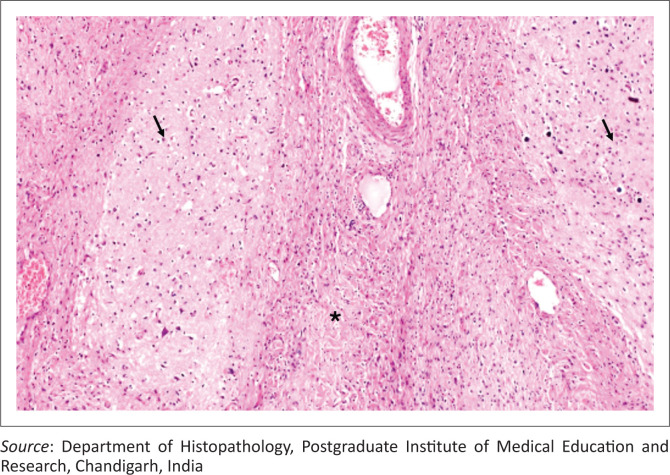
Micrograph demonstrating mature glial tissue (arrows) intermixed with fibroconnective tissue (asterisk) (H&E: Hematoxylin and Eosin. 100×).

As per the institutional preoperative protocol, contrast-enhanced CT of the neck was performed to further delineate the lesion extent, airway compromise and relationship with adjacent vascular and aerodigestive structures. It revealed a multiseptated cystic lesion in the left side of the neck with medial extension into the parapharyngeal space, nasopharynx, oropharynx and larynx, and marked airway compromise (Figure 3). In view of worsening respiratory difficulty and progressive increase in size of the neck mass, gross total excision of the mass was undertaken. Histopathological examination of the postoperative biopsy specimen demonstrated mature glial tissue interspersed within fibroconnective stroma, thereby confirming the diagnosis of neuroglial heterotopia (Figure 4). Post-resection follow-up noted no complications, and the patient recovered well.

## Discussion

Neuroglial heterotopia is a rare, benign congenital lesion and is not a true neoplasm. It shows no intracranial communication, unlike encephalocoele. The proposed pathogenesis involves herniation or descent of developing brain tissue through a transient skull base defect, followed by subsequent closure of the defect, resulting in sequestration of heterotopic neuroglial tissue. Other proposed mechanisms include abnormalities of anterior neuropore closure and aberrant migration or entrapment of olfactory glial cells.

The nasal cavity remains the most common site, whereas pharyngeal and parapharyngeal lesions are distinctly uncommon. Published case reports and case series suggest that parapharyngeal lesions frequently present in infancy with respiratory distress, feeding difficulty, failure to thrive or a cervical mass, reflecting their compressive effect on the upper aerodigestive tract.^8^ Some reports also describe associated craniofacial or congenital anomalies, including cleft palate, Pierre Robin sequence and congenital heart disease.^9^

Neuroglial heterotopia is composed of neuroglial elements but may contain elements of the choroid plexus. Growth of the heterotopic brain tissue in the mass will parallel that of normal brain tissue. Cystic components within the mass are the result of excessive CSF production by the ectopic choroid plexus.

Clinical presentation is dependent on the size of the mass and compression of adjacent structures. Patients usually present with an enlarging neck mass, respiratory difficulty and failure to thrive. Radiological diagnosis is challenging because there are no pathognomonic imaging findings. These lesions are often initially misinterpreted as cystic hygroma or lymphatic malformation, particularly when they contain a substantial cystic component, as in the present case. The large amount of CSF produced by the choroid plexus expands the small heterotopic brain tissue.^10,11^

Cross-sectional imaging is nevertheless crucial because MRI and/or CT can define lesion extent, demonstrate skull base remodelling or adjacent bony displacement when present, assess airway compromise, and most importantly, MRI can exclude intracranial communication. On MRI, neuroglial heterotopia typically shows signal characteristics similar to brain parenchyma in the solid component, while cystic areas may be present as described in this case report. Cystic areas will have signal attenuation similar to that of CSF. Peripheral or mild enhancement may be seen depending on the amount of fibrous stroma and vascularity. The post-contrast enhancement of the wall can be explained by dense fibrosis of the wall, which also contains choroid plexus. In the present case, a small central focus of intrinsic T1 hyperintensity demonstrated signal suppression on the fat-suppressed sequence, suggesting a tiny fat-containing component. Although fat is not a typical dominant imaging feature of neuroglial heterotopia, rare reports have described scattered adipose tissue within glial heterotopia.^12^ Therefore, this finding should be interpreted as an associated minor component rather than a defining feature.

Histologically, the lesions are composed of a variety of elements, including astrocytes, oligodendrocytes, neurons or functioning choroid plexus, interspersed in fibrous tissue.^13^ In the presence of significant fibrosis, glial cells are identified by glial fibrillary acidic protein (GFAP) and S-100 proteins.^14^ Neoplasms like oligodendrogliomas and astrocytoma have also been reported.^15,16^ Definitive diagnosis rests on histopathology, because preoperative distinction from other congenital lesions is difficult on imaging alone.

The differential diagnosis of a cervical mass in an infant is broad and includes encephalocoeles, meningocoeles, cystic hygromas and teratomas. Encephalocoele and meningocoele distinctly demonstrate intracranial communication in contradistinction to heterotopias. Teratomas have many additional components, including fat and calcifications, in addition to the cystic component. Cystic schwannomas are very rare in neonates and primarily arise in the parapharyngeal space, where ectopic neural cells can be found.

Complete surgical excision remains the treatment of choice. Surgery is usually undertaken because of airway compromise, feeding difficulty, progressive enlargement or uncertainty in diagnosis.^17,18^ The timing of surgery is usually controversial, as neonatal procedures are difficult because of the surgical plane. Prognosis is generally favourable after complete resection, although recurrence can occur after incomplete excision; therefore, clinical follow-up is advisable. In the presented patient, surgery was warranted because of progressive respiratory compromise, and histopathology provided the final diagnosis after a preoperative radiologic impression of cystic hygroma.

## Conclusion

Parapharyngeal neuroglial heterotopia should be considered in the differential diagnosis of congenital lateral neck masses in infants, especially in the presence of a complex cystic lesion extending towards the parapharyngeal space associated with airway obstruction. Although preoperative diagnosis is difficult, careful MRI assessment to exclude intracranial communication and timely surgical excision are central to management. Histopathological evaluation remains essential for definitive diagnosis.

## References

[1] Broniatowski M, Witt WJ, Shah AC, Galloway PG, Abramowsky CR. Glial tissue in the parapharyngeal space. Arch Otolaryngol. 1981;107(10):638–641. 10.1001/archotol.1981.00790460050016 7283831

[2] Low NL, Scheinberg L, Andersen DH. Brain tissue in the nose and throat. Pediatrics. 1956;18(2):254–259. 10.1542/peds.18.2.254 13349339

[3] Alsayid HA, Alnoury IS. Parapharyngeal neuroglial heterotopia: A case report and literature review. Am J Case Rep. 2020;21:e926300. 10.12659/AJCR.926300 33156818 PMC7656088

[4] Elder JE, Chow CW, Holmes AD. Heterotopic brain tissue in the orbit: Case report. Br J Ophthalmol. 1989;73(11):928. 10.1136/bjo.73.11.928 2605150 PMC1041930

[5] Zook EG, Nickey WM, Pribaz JJ, Zonk EG. Heterotopic brain tissue in the scalp. Plast Reconstr Surg. 1984;73(4):660–663. 10.1097/00006534-198404000-00026 6709747

[6] Kallman JE, Loevner LA, Yousem DM, et al. Heterotopic brain in the pterygopalatine fossa. Am J Neuroradiol. 1997;18(1):176–179. 9010538 PMC8337871

[7] Chen D, Dedhia K, Ozolek J, Mehta D. Case series of congenital heterotopic neuroglial tissue in the parapharyngeal space. Int J Pediatr Otorhinolaryngol. 2016;86:77–81. 10.1016/j.ijporl.2016.04.026 27260585

[8] Chen CY, Huang JH, Choi WM, Chen CL, Chan WP. Parapharyngeal neuroglial heterotopia presenting as a growing single locular cyst: MR imaging findings. AJNR Am J Neuroradiol. 2005;26(1):96–99. 15661709 PMC7975011

[9] Longo D, Menchini L, Delfino LN, et al. Parapharyngeal neuroglial heterotopia in Pierre Robin sequence: MR imaging findings. Int J Pediatr Otorhinolaryngol. 2009;73(9):1308–1310. 10.1016/j.ijporl.2009.05.014 19540602

[10] Fuller C, Gibbs AR. Heterotopic brain tissue in the lung causing acute respiratory distress in an infant. Thorax. 1989;44(12):1045–1046. 10.1136/thx.44.12.1045 2694419 PMC1020883

[11] Wismer GL, Wilkinson Jr AH, Goldstein JD. Cystic temporofacial brain heterotopia. AJNR Am J Neuroradiol. 1989;10(5 suppl.):S32–S33. 2505556 PMC8333923

[12] Xian J. Computed tomographic and magnetic resonance imaging findings in the midline nasopharyngeal glial heterotopia. Austin J Radiol. 2015;2(2):1015.

[13] Gyure KA, Thompson LD, Morrison AL. A clinicopathological study of 15 patients with neuroglial heterotopias and encephaloceles of the middle ear and mastoid region. Laryngoscope. 2000;110(10):1731–1735. 10.1097/00005537-200010000-00032 11037835

[14] Behar PM, Muller S, Gerber ME, Todd NW. Heterotopic neuroglial tissue causing airway obstruction in the newborn. Arch Otolaryngol Neck Surg. 2001;127(8):997–1002. 10.1001/archotol.127.8.997 11493213

[15] Lee SC, Henry MM, Gonzalez-Crussi F. Simultaneous occurrence of melanotic neuroectodermal tumor and brain heterotopia in the oropharynx. Cancer. 1976;38(1):249–253. 10.1002/1097-0142(197607)38:1<249::AID-CNCR2820380137>3.0.CO;2-D 181129

[16] Bossen EH, Hudson WR. Oligodendroglioma arising in heterotopic brain tissue of the soft palate and nasopharynx. Am J Surg Pathol. 1987;11(7):571–574. 10.1097/00000478-198707000-00010 3605491

[17] Huisman TA, Brehmer U, Zeilinger G, Stallmach T, Gysin C. Parapharyngeal neuroglial heterotopia extending through the skull base in a neonate with airway obstruction. J Pediatr Surg. 2007;42(10):1764–1767. 10.1016/j.jpedsurg.2007.06.014 17923212

[18] Okeda R. Heterotopic brain tissue in the submandibular region and lung: Report of two cases and comments about pathogenesis. Acta Neuropathol (Berl). 1978;43(3):217–220. 10.1007/BF00691581 696239

